# Utility of PET/CT in the Diagnosis and Staging of Extranodal Natural Killer/T-Cell Lymphoma: A Systematic Review and Meta-Analysis

**DOI:** 10.1097/MD.0000000000000258

**Published:** 2014-12-02

**Authors:** Xiangxiang Zhou, Kang Lu, Lingyun Geng, Xinyu Li, Yujie Jiang, Xin Wang

**Affiliations:** From the Department of Hematology, Shandong Provincial Hospital Affiliated to Shandong University, Jinan, Shandong 250021 (XZ, KL, LG, XL, YJ, XW); and Department of Diagnostics, Shandong University School of Medicine, Jinan, Shandong 250012, P.R. China (XW).

## Abstract

The role of ^18^F-fuorodexoyglucose-positron emission tomography/computed tomography (^18^F-FDG-PET/CT) in the staging of Hodgkin lymphoma (HL) and aggressive B-cell non-Hodgkin lymphomas (NHL) has been demonstrated extensively. Nevertheless, the role of PET/CT in the diagnosis, staging, prognosis, and treatment evaluation of natural killer (NK)/T-cell lymphoma remains indeterminate.

To systematically review and meta-analyze the publications on the value of ^18^F-FDG-PET/CT in the diagnosis and staging of NK/T-cell lymphoma.

Pubmed, Embase, Cochrane Library, and some other database were searched for initial studies (last updated on May 8th, 2014).

The eligibility criteria were studies assessing the usefulness of PET/CT in the staging of NK/T-cell lymphoma, patients were diagnosed as NK/T-cell lymphoma through pathology, or clinical and imaging follow-up.

Sensitivities and specificities of ^18^F-FDG-PET/CT in individual studies were assessed. The summary receiver operating characteristic curve (sROC) and the area under the curve (AUC) were calculated. The “Meta-Disc 1.4” software was used for data analysis.

Eight studies, with a total of 135 NK/T-cell lymphoma patients, were included in this meta-analysis. In terms of the 6 studies with patient based data, the pooled sensitivity and specificity of PET/CT in the diagnosis of NK/T-cell lymphoma were 0.95 (95% CI: 0.89–0.98) and 0.40 (95% CI: 0.09–0.78), respectively. For lesion-based analysis, with 1546 lesions included, the pooled sensitivity and specificity of PET/CT in the staging of NK/T-cell lymphoma were 0.98 (95% CI: 0.96–0.99) and 0.99 (95% CI: 0.99–1.00), respectively. For the patient based data, the AUC and ^∗^Q index were 0.8537 and 0.7847, respectively. For lesion based data, the AUC and ^∗^Q index were 0.9959 and 0.9755, respectively.

The results of this current meta-analysis indicated that PET/CT could be used as a valuable diagnostic and staging tool for NK/T-cell lymphoma.

## INTRODUCTION

Extranodal natural killer (NK)/T-cell lymphoma (ENKTL) is a newly recognized distinctive entity in the World Health Organization (WHO) classification of lymphomas.^1^ It accounts for less than 1% of all lymphomas in Western countries and approximately 3% to 9% of all lymphomas in Asia.^2–4^ ENKTL is an aggressive lymphoma with poor survival outcome and the cumulative probability of 5-year survival ranging from 37.9% to 49.5%.^5^ However, optimal treatment strategies have not been identified. Accurate staging plays a decisive role in the prognosis and treatment strategy of NK/T-cell lymphoma.^6,7^

^18^F-fuorodexoyglucose-positron emission tomography/computed tomography (^18^F-FDG-PET/CT) is a functional imaging test that has been widely used in the staging, prognosis, and treatment of Hodgkin lymphoma (HL) and various types of B-cell non-Hodgkin lymphomas (NHL).^8–10^ However, similar studies in T-cell and NK-cell lymphomas are relatively rare. Therefore, a systematic review aimed to evaluate the effect of PET/CT in the diagnosis and staging of NK/T-cell lymphoma was urgently needed. In this present study, we undertook a meta-analysis and systematic review to assess the role of ^18^F-FDG-PET/CT in the diagnosis and staging of NK/T-cell lymphoma.

## MATERIALS AND METHODS

### Literature Search

A systematic computer literature search was performed to identify studies assessing the value of PET or PET/CT in the diagnosis and staging of NK/T-cell lymphoma. The Pubmed, Embase, and Cochrane Library databases were searched with the following keywords (“PET” or “positron emission tomography,” “neutral killer/T-cell lymphoma” or “NK/T-cell lymphoma” or “lymphoma”). No start date limit was used. The search was last updated on May 2014. In addition, reference lists from the included studies were also hand searched. Ethical approval was not necessary for this meta-analysis, due to that all analyses were based on the results of previous published studies.

### Inclusion and Exclusion Criteria

The inclusion criteria were: (1) Studies assessing the usefulness of PET/CT in the staging of NK/T-cell lymphoma, patients were diagnosed as NK/T-cell lymphoma through pathology, or clinical and imaging follow-up. (2) Article with the most detailed or the most recent article was chosen if data were presented in more than 1 article. (3) Articles with sufficient data to construct or calculate the true-positive (TP), false-positive (FP), true-negative (TN), false-negative (FN). (4) With full-text articles published in English.

The exclusion criteria were: (1) Publication did not aim to reveal the value of PET/CT in NK/T-cell lymphoma. (2) Publications without original data, such as case reports, letters, congress, comments, and reviews. (3) Vitro studies and animal experiments. (4) Studies with less than 5 patients with NK/T-cell lymphoma enrolled. (5) With less than 9 “yes” responses to the quality assessment of diagnosis accuracy studies (QUADAS).^11^ QUADAS is an evidence-based quality assessment tool used for assess the diagnostic accuracy of studies in systematic reviews.

### Data Extraction and Quality Assessment

Two investigators independently extracted the data needed through screening the abstracts and full-text articles. Any differences were resolved by consensus. The criteria list recommended by the Cochrane Methods Working Group on Systematic Review of Screening and Diagnostic Tests was used. The QUADAS quality assessment tool was used to estimate the quality of the included studies.^11^

### Statistical Analysis

Data on max based standardized uptake value (SUV), TP, TN, FP, and FN were calculated from the original data in the publications. Pool estimates of sensitivity and specificity were analyzed. In addition, the 95% CI was also constructed. The summary receiver operating characteristic curve (sROC) and the area under the curve (AUC) were calculated. sROC is a curve conducted with the sensitivity as y-axis and the “1-specificity” as x-axis. It reflects the continuous variables of sensitivity and specificity. The closer of sROC to the top left corner of coordinate axis, the higher of the accuracy of diagnostic studies. AUC is the area under sROC, which is used to assess the total efficiency of diagnosis. We also estimated the maximum joint sensitivity and specificity (Q index) to measure the overall diagnostic accuracy (the point on the sROC curve at which the sensitivity and specificity are equal). The patient-based data and lesion-based data were conducted, respectively. All data analyze were performed using the Meta-Disc software version 1.4. *P*-value of <0.05 were considered to be statistically significant.

## RESULTS

### Literature Search and Selection of Studies

**FIGURE 1:**
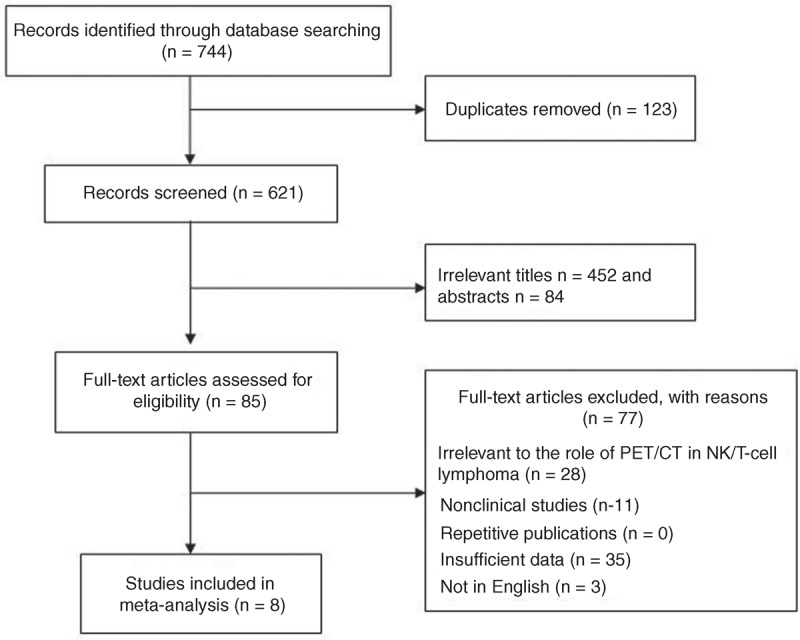
Studies evaluated for inclusion in this meta-analysis.

According to the search strategy, a total of 744 papers were selected: 409 in Pubmed, 335 in Embase, 0 in Cochrane Library, and 0 by hand search (last updated on May 8th, 2014). After reading abstracts, we reviewed 85 studies in detail. Of these articles, 55 were excluded because: (1) Aim of these articles was not to reveal the value of PET/CT in the staging and prognosis of NK/T-cell lymphoma. (2) Insufficient data were reported to construct or calculate the TP, TN, FN, and FP or PFS, OS. (3) The number of “yes” response to the 14 QUADAS questions was less than 9. Finally, 8 studies with 135 patients with NK/T-cell lymphoma were selected for data extraction and analysis (Figure 1).

### Study Design Characteristics and Publication Bias

Data were extracted by 2 authors and any differences were resolved by consensus. Data abstraction was not blinded to authors, institution, or source of publication. Characteristics of the included 8 studies were shown in Table 1. Among them, 6 studies concentrated on the patient based data (Table 2) and 6 studies focused on the lesion based data (Table 3) of the diagnostic value of PET/CT in NK/T-cell lymphoma.

**TABLE 1:**
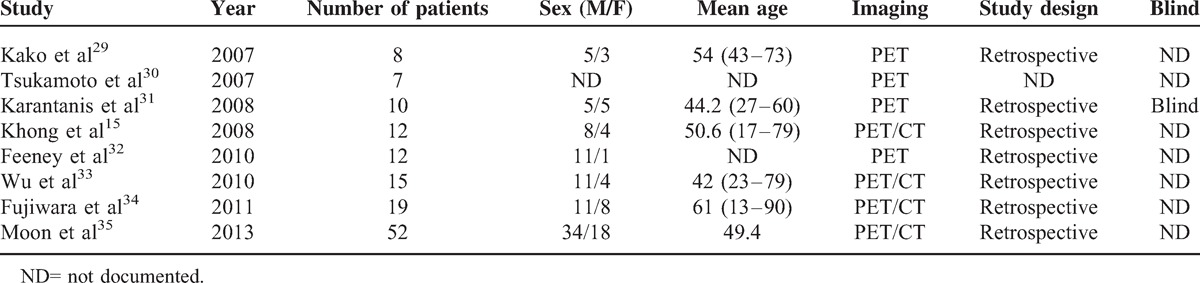
Main Characteristics of the Included Studies

**TABLE 2:**
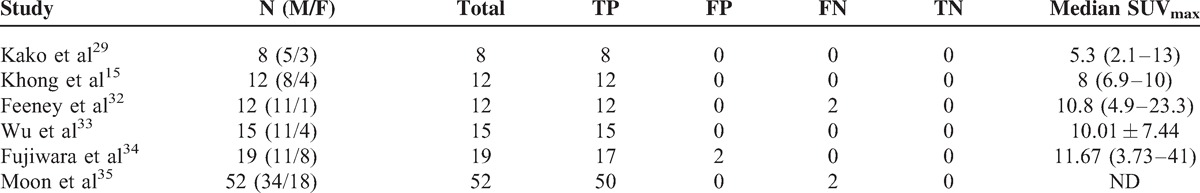
PET/CT in NK/T-Cell Lymphoma: Patient Based Data

**TABLE 3:**
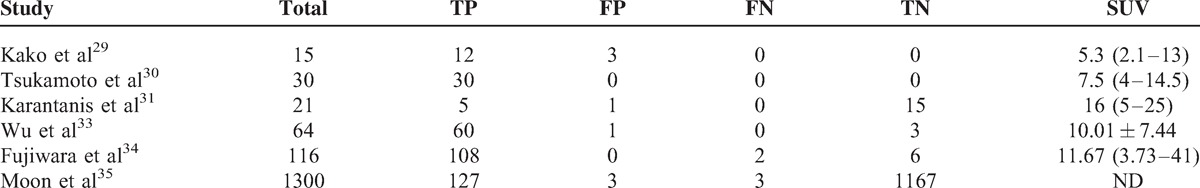
PET/CT in NK/T-Cell Lymphoma: Lesion Based Data

### Summary Estimates of Sensitivity, Specificity, and Diagnostic Odds Ratio

**FIGURE 2:**
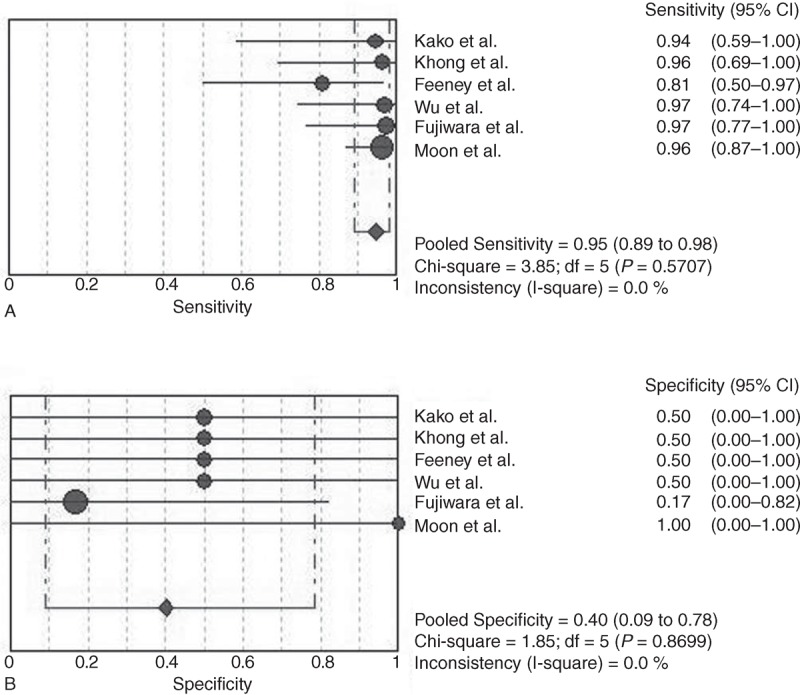
(A) Sensitivity and 95% confidence intervals for studies assessing the diagnostic accuracy of FDG PET in patients with NK/T-cell lymphoma. (B) Specificity and 95% confidence intervals for studies assessing the diagnostic value of PDG PET in patients with NK/T-cell lymphoma. ^∗^The diamond represents the 95% CI of the pooled estimate.

In terms of the 6 studies with patient based data. The Spearman correlation coefficient was 0.507 (*P* = 0.305). These 2 data showed that no threshold effect existed in it. The pooled sensitivity and specificity of PET/CT in the diagnosis of NK/T-cell lymphoma were 0.95 (95% CI: 0.89–0.98) and 0.40 (95% CI: 0.09–0.78), respectively (Figure 2). The pooled positive LR and pooled negative LR were 1.63 (95% CI: 0.91–2.91) and 0.13 (95% CI: 0.05–0.38), respectively. The pooled DOR was 13.28 (95% CI: 2.60–67.82).

**FIGURE 3:**
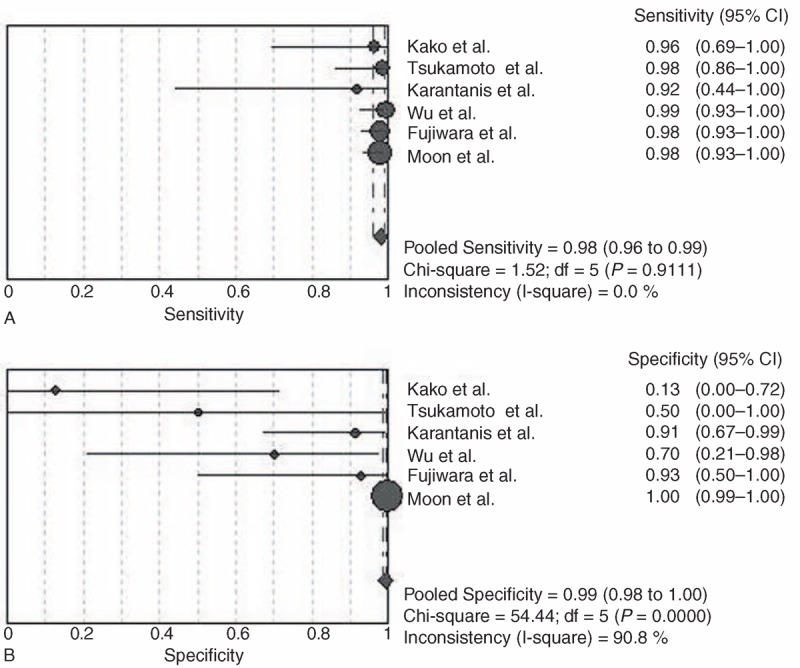
(A) Sensitivity and 95% confidence intervals for studies assessing the diagnostic accuracy of FDG PET with lesion based data. (B) Specificity and 95% CI for studies assessing the diagnostic value of PDG PET with lesion based data. ^∗^The diamond represents the 95% CI of the pooled estimate.

For lesion-based analysis, 6 studies were eligible for meta-analysis. The Spearman correlation coefficient was 0.086 (*P* = 0.872). No threshold effect was found. The pooled sensitivity and specificity of PET/CT in the staging of NK/T-cell lymphoma were 0.98 (95% CI: 0.96–0.99) and 0.99 (95% CI: 0.99–1.00), respectively (Figure 3). The pooled positive LR and pooled negative LR were 8.52 (95% CI: 0.23–312.09) and 0.03 (95% CI: 0.01–0.06), respectively. The pooled DOR was 268.66 (95% CI: 16.8–4296.47).

### Heterogeneity Assessing

The presences of heterogeneity between studies were examined using the chi-square test. No heterogeneity was found in the sensitivity (Chi-square = 3.80, *P* = 0.5784) and specificity (Chi-square = 1.85, *P* = 0.8699) of PET/CT in the patients based data.

### Summary ROC Curves and the ^∗^Q Index

**FIGURE 4:**
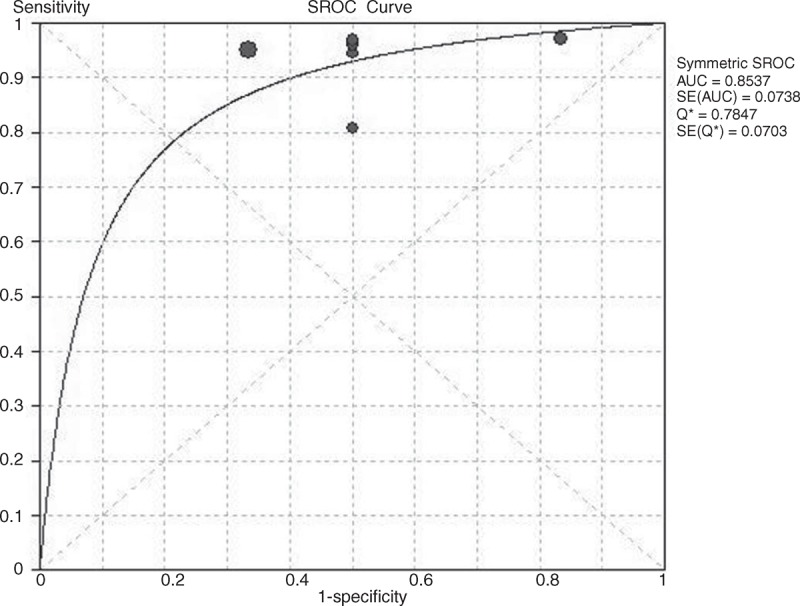
The SROC curves of PET/CT in the patient-based data of NK/T-cell lymphoma.

**FIGURE 5:**
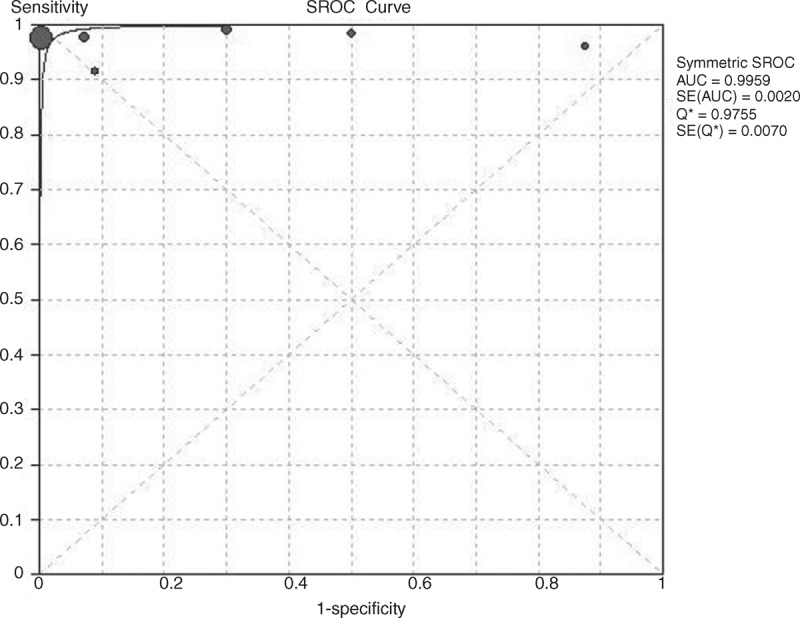
The SROC curves of PET/CT in the lesion-based data of NK/T-cell lymphoma.

The SROC curves and the ^∗^Q index for PET/CT in the diagnosis and staging of NK/T-cell lymphoma were shown in Figures 4 and 5. In the data based on patients, no heterogeneity and threshold effect were found. Therefore, we chose the fixed-effects model (Mantel–Haenszel) to analysis the SROC curves. The results showed that the AUC and ^∗^Q index were 0.8537 and 0.7847, respectively. For lesion-based data, the difference between b and 0 (*P* = 0.0132) is of statistical significance. Therefore, the Moses–Sphaprio–Littenberg method was used to construct the sROC. The AUC and ^∗^Q index were 0.9959 and 0.9755, respectively. This indicated that PET/CT is of great value in the detecting the lesions of NK/T-cell lymphoma.

## DISCUSSION

The role of PET/CT in the staging of HL and aggressive B-cell NHL has been demonstrated extensively. Recently, several clinical studies have investigated the utility of PET/CT in the NK/T-cell lymphoma with some promising results in the diagnostic value of PET/CT in NK/T-cell lymphoma.^12–14^ Nevertheless, the role of PET/CT in the diagnosis, staging, prognosis, and treatment evaluation of NK/T-cell lymphoma remains indeterminate.

Due to the rarity of NK/T-cell lymphoma, limited investigations have been carried out in the diagnosis and staging of NK/T-cell lymphoma. Khong et al reported that the lesions of NK/T-cell lymphoma were FDG-PET avid in 2008.^15^ It was then indicated that the PET/CT was true positive in 5 cases, true negative in 15 cases, and 1 case unconfirmed of the total 21 lesions examined. The mean SUV(max) was relatively higher (16 g/mL) in nasal cavities than that in extranasal cases (10.9 g/mL).^16^ Furthermore, Suh et al identified that high FDG uptake was related to poor treatment responses and outcomes.^17^ It was recently suggested that PET/CT plays a more important role than MRI or PET alone in the staging of lymphoma.^18^ But, those studies were limited due to the low incidence of NK/T-cell lymphoma. The utility of PET/CT in the diagnosis and staging of this lymphoma is still unclear. Efforts are still needed to improve the staging accuracy of NK/T-cell lymphoma.

This systematic review and meta-analysis focused on evaluating the utility of PET/CT in the diagnosis and staging of NK/T-cell lymphoma. Eight studies with a total of 135 patients with NK/T-cell lymphoma were included. The results of this meta-analysis indicated that PET/CT has a high diagnostic accuracy in the evaluation of the staging in patients with NK/T-cell lymphoma. The pooled sensitivity was 0.95 and the pooled specificity was 0.4 in the patient based analysis. Moreover, the pooled positive and negative LR was 1.63 and 0.13, respectively. In the patient based data, the AUC was 0.8537 and the ^∗^Q was 0.7847. This illustrated that PET/CT is valuable in the diagnosis of NK/T-cell lymphoma. This conclusion was consistent with previous studies. However, the specificity is not very optimal in patient based data. On the other hand, we analyzed lesion-based data in patients with NK/T-cell lymphoma. The pooled sensitivity and the pooled specificity were 0.98 and 0.99, respectively. Moreover, the pooled DOR, the AUC and ^∗^Q index were 268.66, 0.9959, and 0.9755, respectively. The results demonstrated that PET/CT presented high sensitivity and specificity in detecting the NK/T-cell lymphoma related lesions. On the other hand, significant higher diagnostic accuracy was found in the lesion based analysis compared to that in the patient based analysis.

In the clinical practice, the treatment strategies of NK/T-cell lymphoma patients depend on the stage of disease. Patients with stage I/II NK/T-cell lymphoma showed more favorable prognosis than that with stage III/IV disease.^19,20^ Radiotherapy, sometimes combined with chemotherapy, is the main treatment strategy for stage I/II NK cell lymphomas.^21,22^ However, due to the highly aggressive and refractory of III/IV stage NK/T-cell lymphoma, chemotherapy acts as the mainstay choice of treatment. Most recently, the l-asparaginase based chemotherapeutic regimens, such as SMILE, have been proved to be effective in the treatment of advanced, relapsed, or refractory NK/T-cell lymphomas.^23,24^ In patients with advanced NK/T-cell lymphoma, this regimen showed a complete remission rate of 35% to 50% and an overall response rate of 74%.^24^ Nevertheless, due to the rarity of NK/T-cell lymphoma, the best treatment strategy is still unclear.

The prognostic role of International Prognostic Index (IPI) in NK/T-cell lymphoma has been validated in several studies.^25^ Recently, Glasgow Prognostic Score (GPS), a novel prognostic score based on CRP and albumin levels, was found to be superior to IPI in the prognosis of ENKL.^26^ But the IPI has also failed to predict survival in patients with ENKTCL.^27^ It has been revealed that PET/CT is a promising tool to diagnostic and staging of NK/T-cell lymphoma. Several investigations identified that almost all NK/T-cell lymphomas are FDG avid.^15,28^ Therefore, PET/CT represents a valuable addition to the staging procedures. Because of the rarity of NK/T-cell lymphoma, the role of PET/CT in the diagnosis and staging of NK/T-cell lymphoma is still indeterminate yet. Our results provided an analysis of the diagnostic performance of PET/CT in NK/T-cell lymphoma.

Nevertheless, there are several potential limitations during the meta-analysis conduction of diagnostic tests. Due to the limitations of the existing literature, further studies are needed to confirm the value of PET/CT in the prediction and treatment assessment of NK/T-cell lymphoma. In addition, other factors such as the retrospective design of studies and heterogeneity, are also involved in the limitations of this meta-analysis.

## CONCLUSIONS

The findings from this meta-analysis demonstrate that PET/CT is a valuable tool in the diagnosis and staging for NK/T-cell lymphoma. However, it was not determined with certainty whether PET/CT is useful in the treatment response of NK/T-cell lymphoma. Furthermore, new parameters of PET should also be considered in the diagnosis of lymphoma. It is hopeful to add PET/CT to the routine staging workup of lymphoma.
